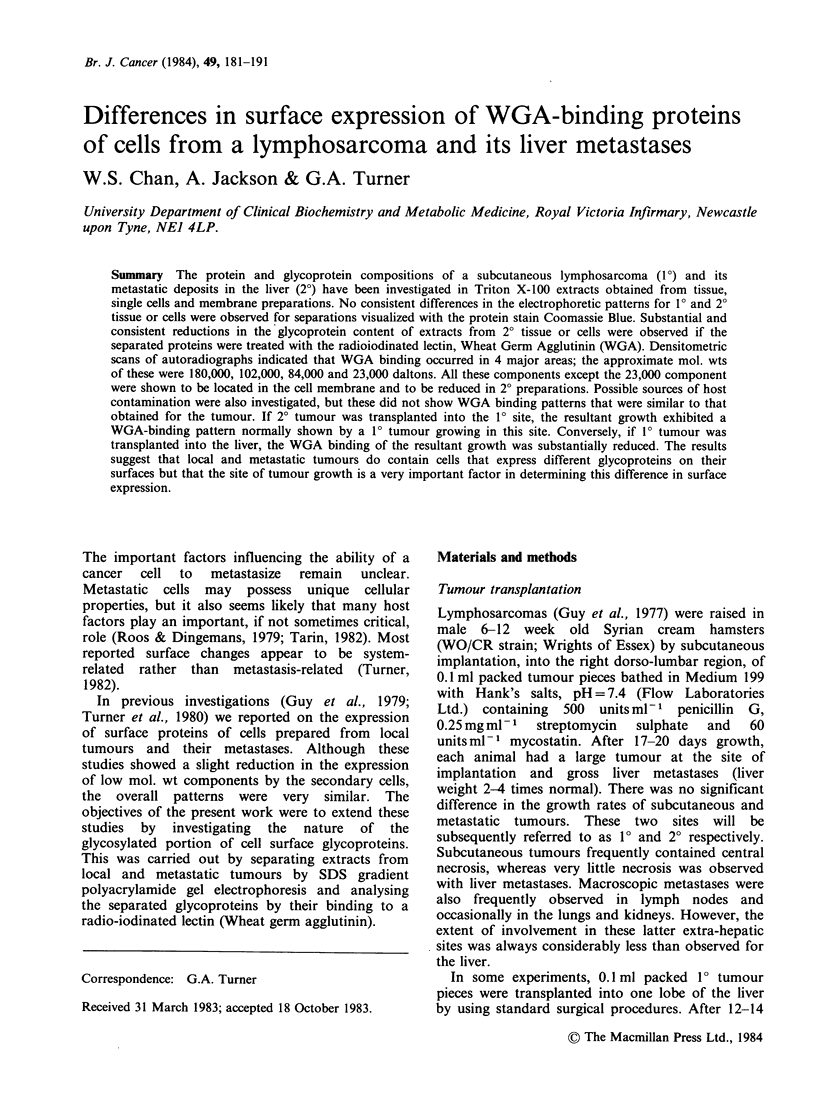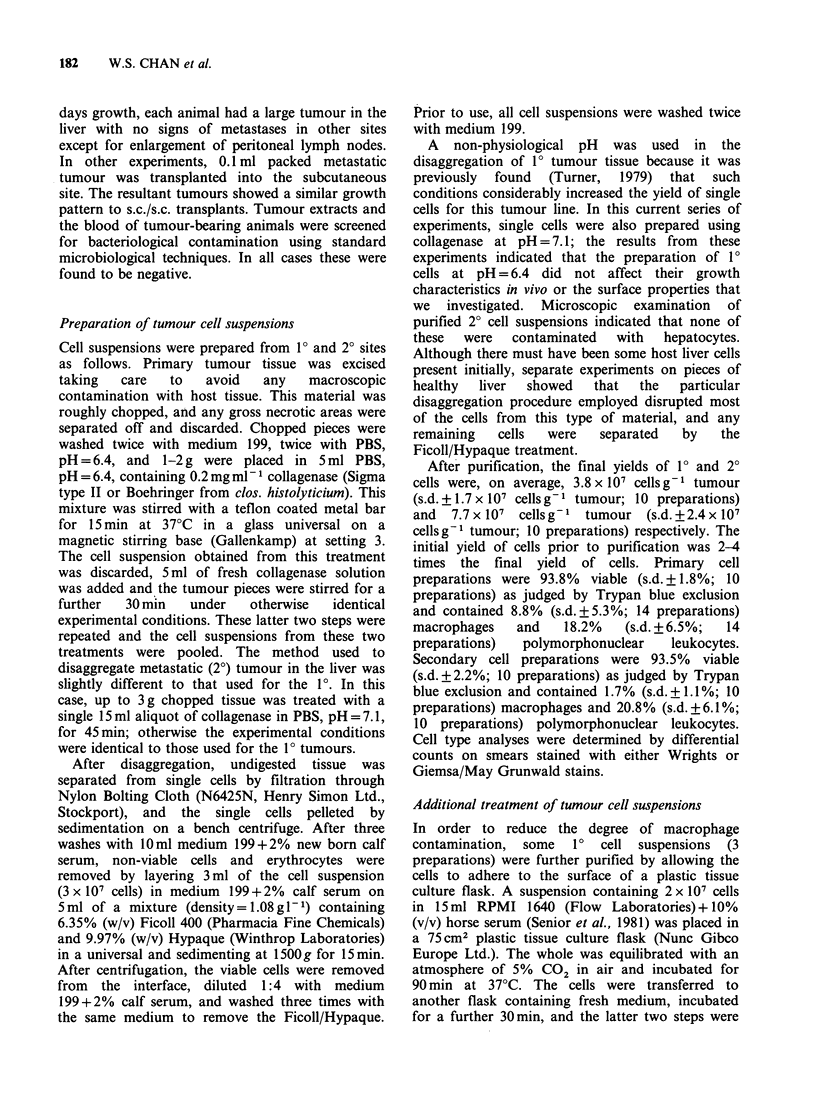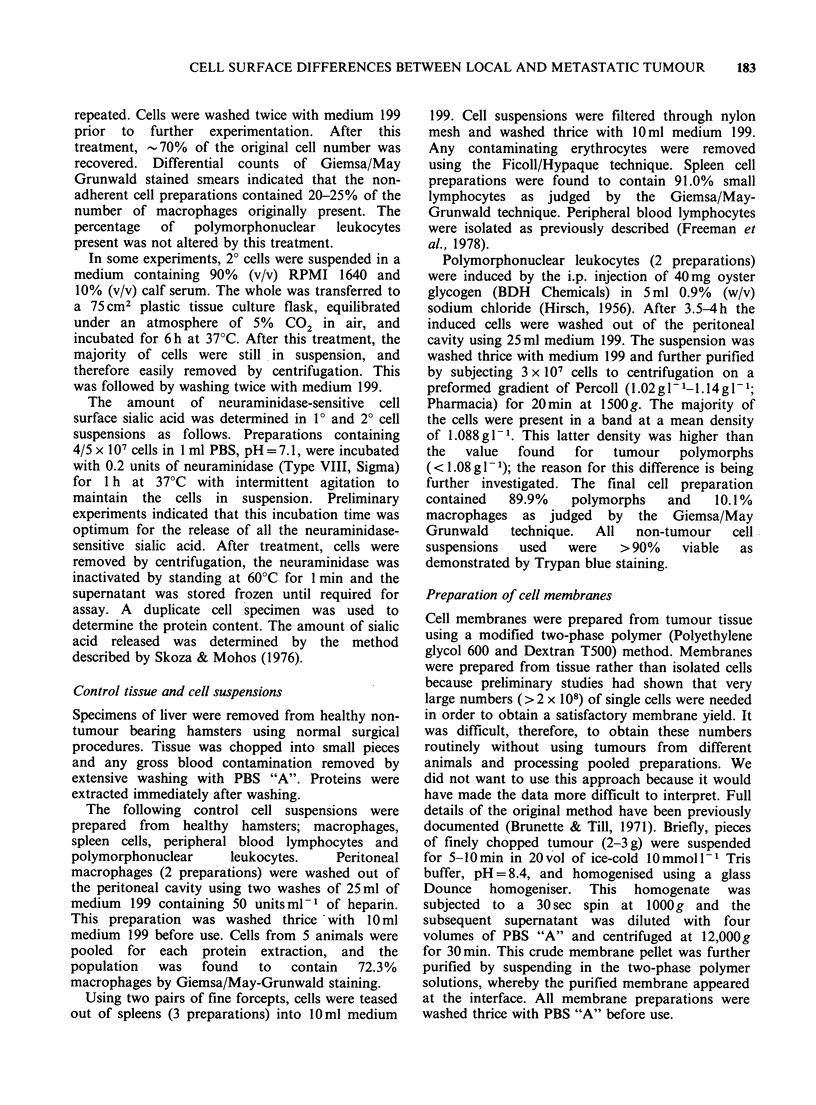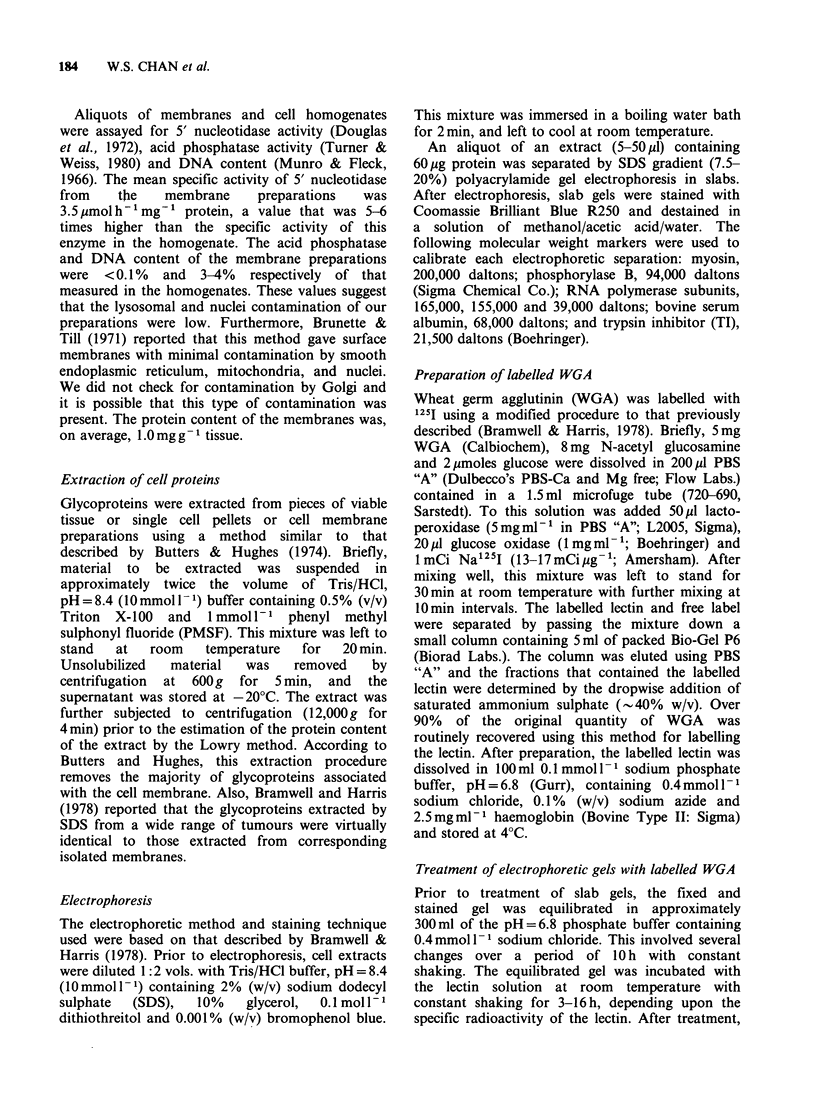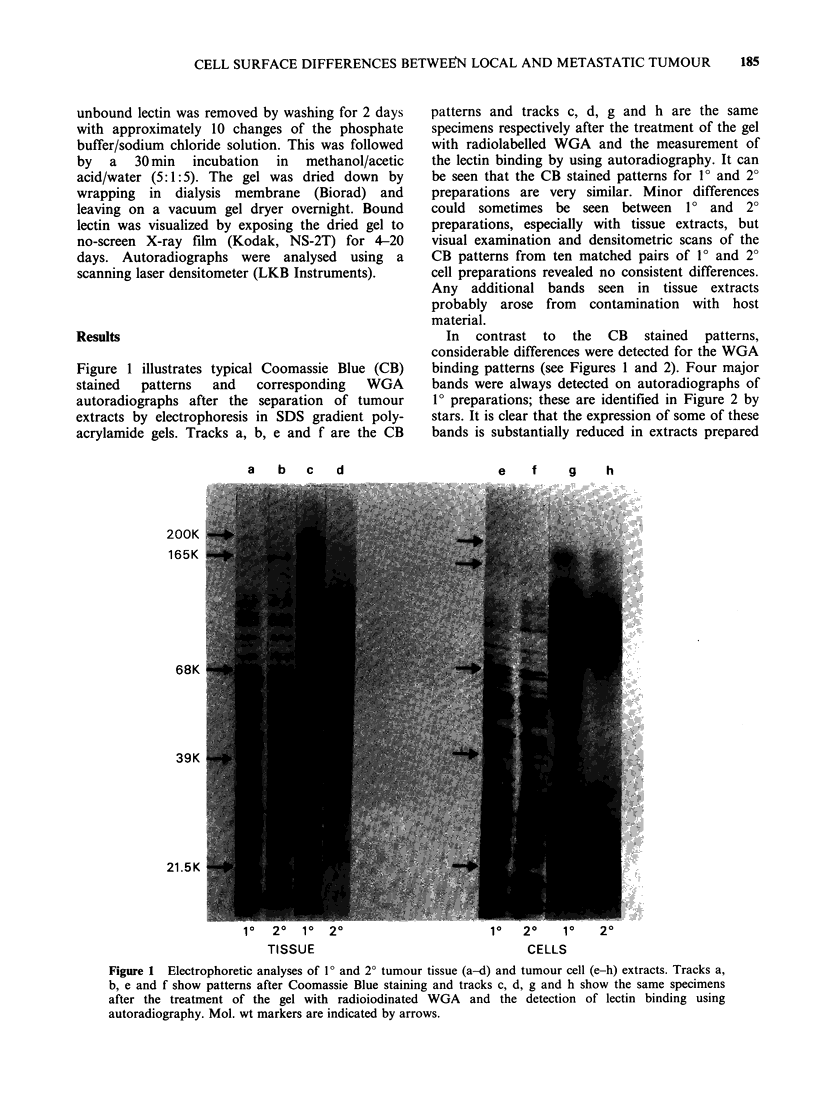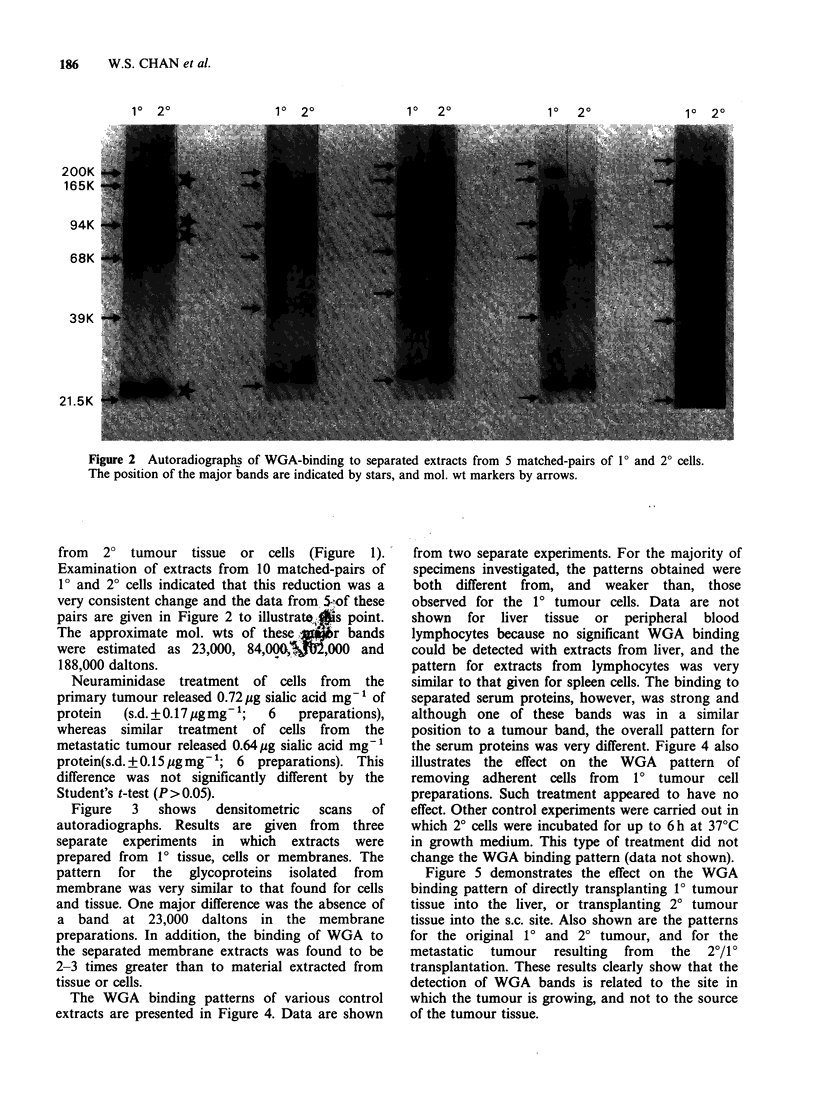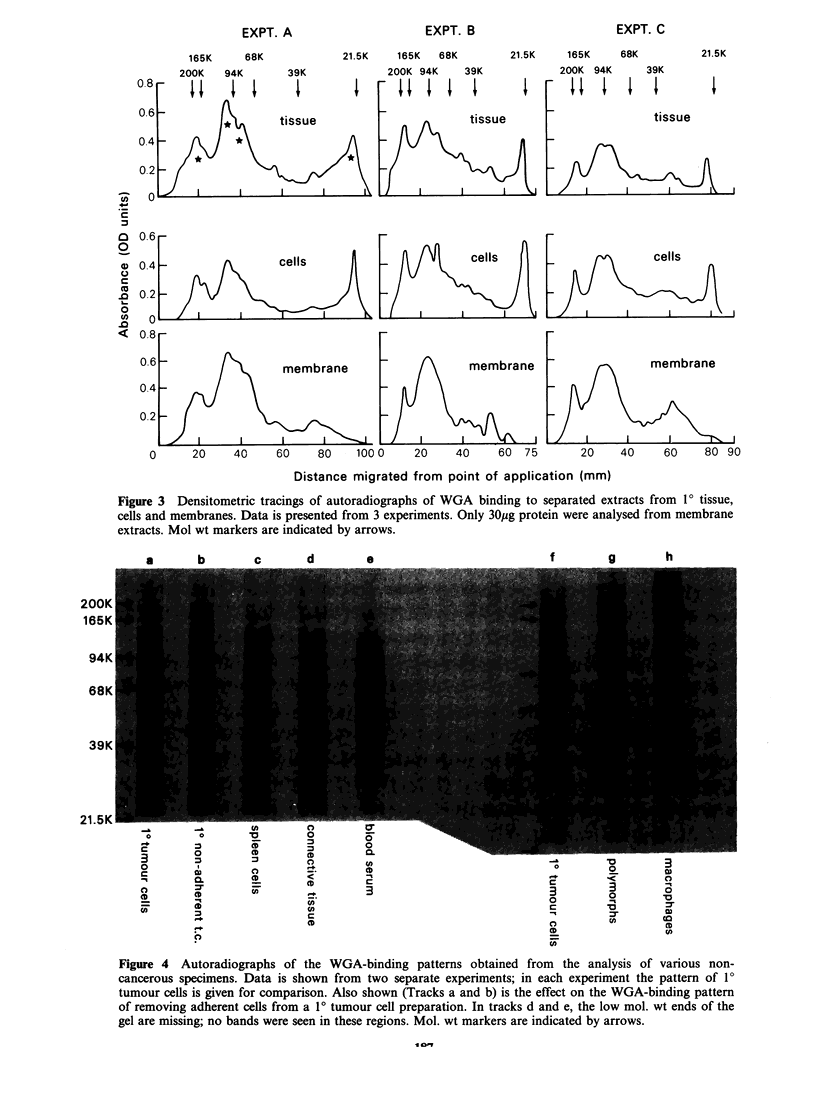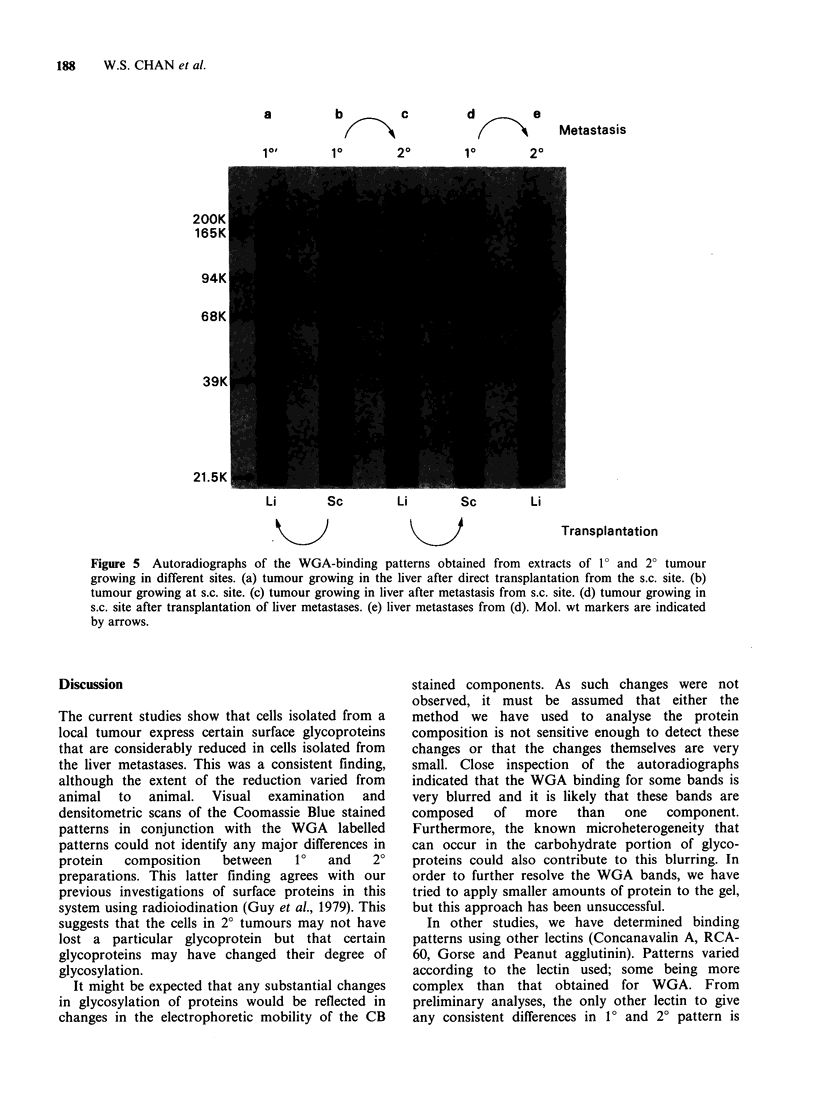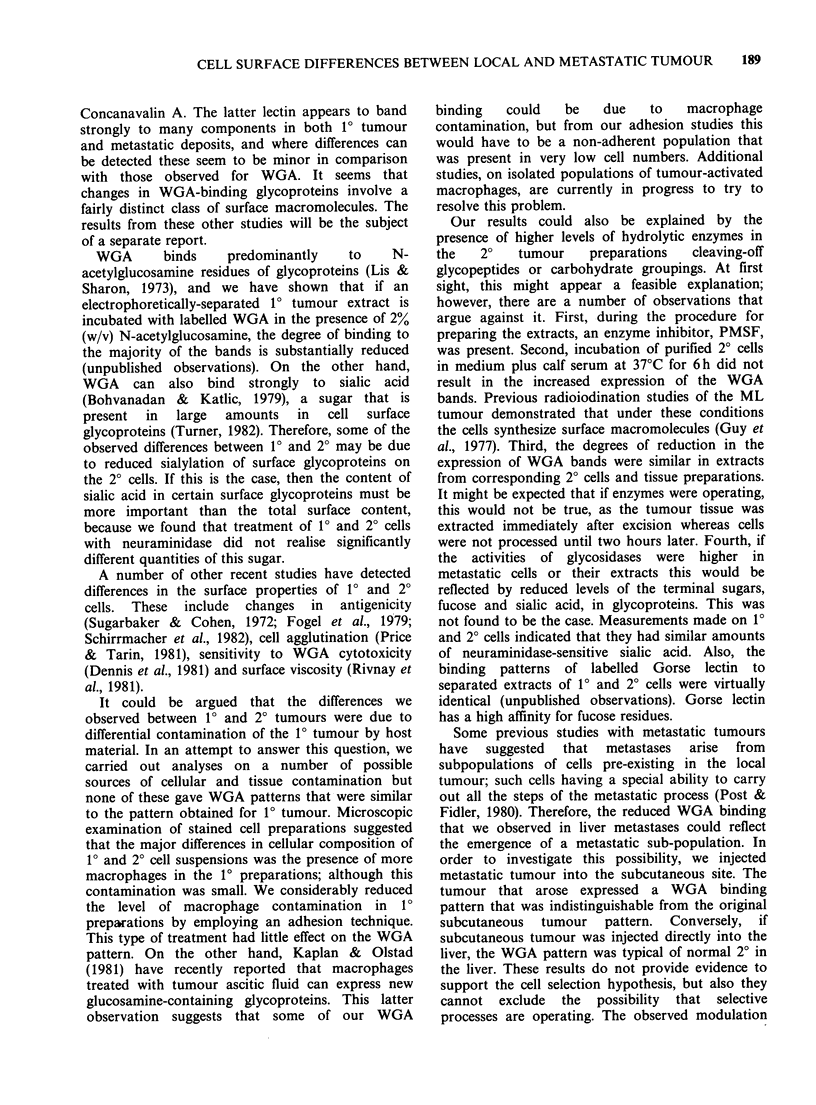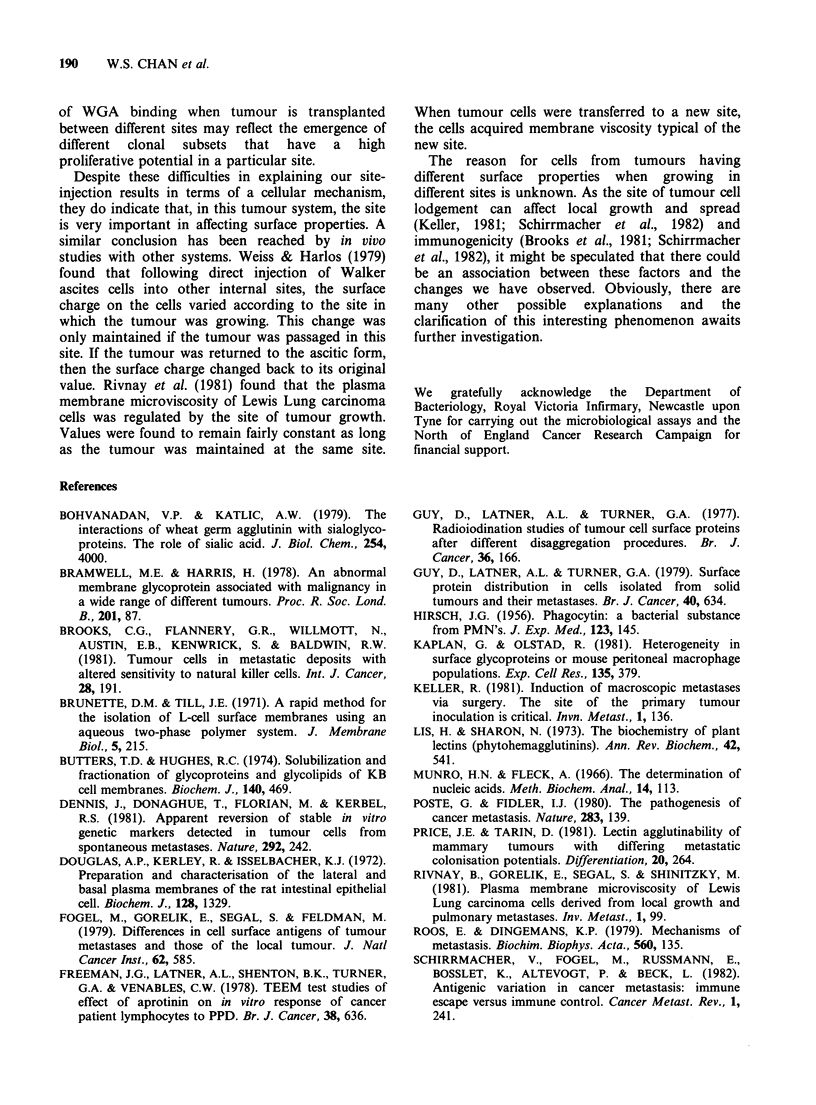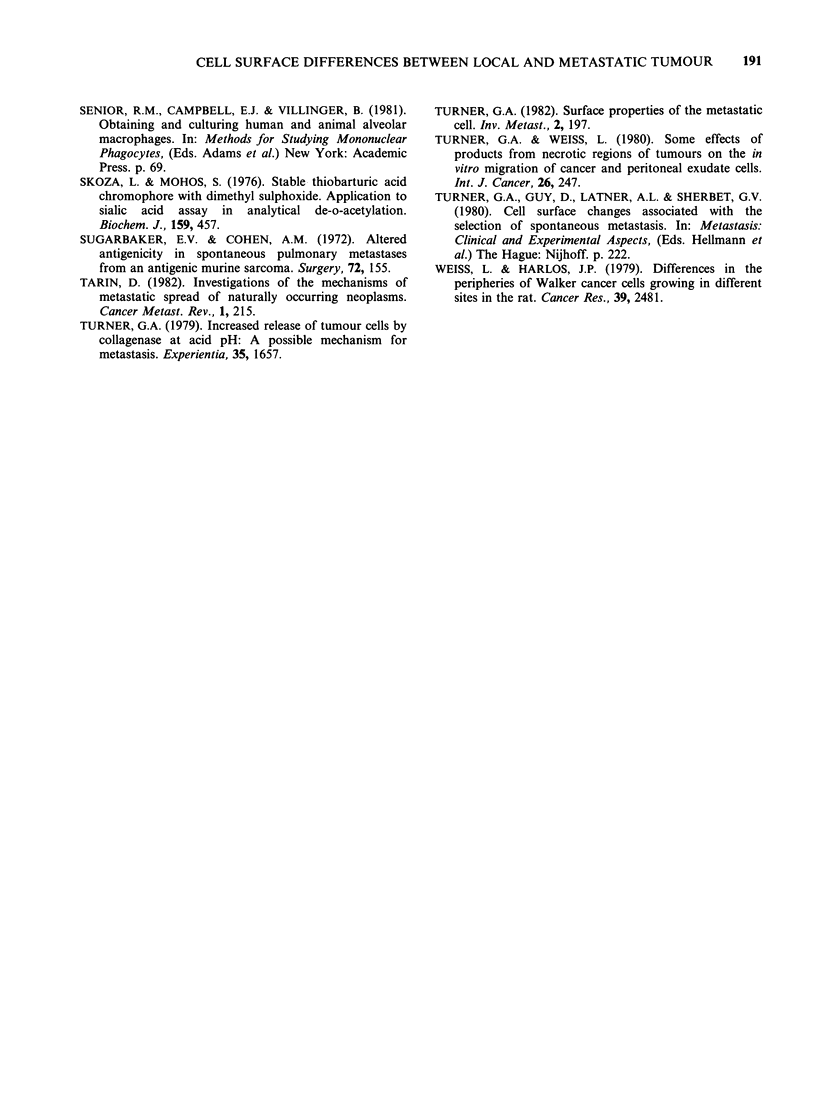# Differences in surface expression of WGA-binding proteins of cells from a lymphosarcoma and its liver metastases.

**DOI:** 10.1038/bjc.1984.30

**Published:** 1984-02

**Authors:** W. S. Chan, A. Jackson, G. A. Turner

## Abstract

**Images:**


*Br. J. Cancer* (1984), **49**, 181–191

### Differences in surface expression of WGA-binding proteins of cells from a lymphosarcoma and its liver metastases

W.S. Chan, A. Jackson & G.A. Turner

*University Department of Clinical Biochemistry and Metabolic Medicine, Royal Victoria Infirmary, Newcastle upon Tyne, NE1 4LP.*

**Summary** The protein and glycoprotein compositions of a subcutaneous lymphosarcoma (1°) and its metastatic deposits in the liver (2°) have been investigated in Triton X-100 extracts obtained from tissue, single cells and membrane preparations. No consistent differences in the electrophoretic patterns for 1° and 2° tissue or cells were observed for separations visualized with the protein stain Coomassie Blue. Substantial and consistent reductions in the glycoprotein content of extracts from 2° tissue or cells were observed if the separated proteins were treated with the radioiodinated lectin, Wheat Germ Agglutinin (WGA). Densitometric scans of autoradiographs indicated that WGA binding occurred in 4 major areas; the approximate mol. wts of these were 180,000, 102,000, 84,000 and 23,000 daltons. All these components except the 23,000 component were shown to be located in the cell membrane and to be reduced in 2° preparations. Possible sources of host contamination were also investigated, but these did not show WGA binding patterns that were similar to that obtained for the tumour. If 2° tumour was transplanted into the 1° site, the resultant growth exhibited a WGA-binding pattern normally shown by a 1° tumour growing in this site. Conversely, if 1° tumour was transplanted into the liver, the WGA binding of the resultant growth was substantially reduced. The results suggest that local and metastatic tumours do contain cells that express different glycoproteins on their surfaces but that the site of tumour growth is a very important factor in determining this difference in surface expression.

The important factors influencing the ability of a cancer cell to metastasize remain unclear. Metastatic cells may possess unique cellular properties, but it also seems likely that many host factors play an important, if not sometimes critical, role (Roos & Dingemans, 1979; Tarin, 1982). Most reported surface changes appear to be system-related rather than metastasis-related (Turner, 1982).

In previous investigations (Guy *et al.*, 1979; Turner *et al.*, 1980) we reported on the expression of surface proteins of cells prepared from local tumours and their metastases. Although these studies showed a slight reduction in the expression of low mol. wt components by the secondary cells, the overall patterns were very similar. The objectives of the present work were to extend these studies by investigating the nature of the glycosylated portion of cell surface glycoproteins. This was carried out by separating extracts from local and metastatic tumours by SDS gradient polyacrylamide gel electrophoresis and analysing the separated glycoproteins by their binding to a radio-iodinated lectin (Wheat germ agglutinin).

Correspondence: G.A. Turner

Received 31 March 1983; accepted 18 October 1983.

### Materials and methods

### *Tumour transplantation*

Lymphosarcomas (Guy *et al.*, 1977) were raised in male 6–12 week old Syrian cream hamsters (WO/CR strain; Wrights of Essex) by subcutaneous implantation, into the right dorso-lumbar region, of 0.1 ml packed tumour pieces bathed in Medium 199 with Hank's salts, pH = 7.4 (Flow Laboratories Ltd.) containing 500 units $ml^{-1}$ penicillin G, 0.25 mg $ml^{-1}$ streptomycin sulphate and 60 units $ml^{-1}$ mycostatin. After 17–20 days growth, each animal had a large tumour at the site of implantation and gross liver metastases (liver weight 2–4 times normal). There was no significant difference in the growth rates of subcutaneous and metastatic tumours. These two sites will be subsequently referred to as 1° and 2° respectively. Subcutaneous tumours frequently contained central necrosis, whereas very little necrosis was observed with liver metastases. Macroscopic metastases were also frequently observed in lymph nodes and occasionally in the lungs and kidneys. However, the extent of involvement in these latter extra-hepatic sites was always considerably less than observed for the liver.

In some experiments, 0.1 ml packed 1° tumour pieces were transplanted into one lobe of the liver by using standard surgical procedures. After 12–14

© The Macmillan Press Ltd., 1984

days growth, each animal had a large tumour in the liver with no signs of metastases in other sites except for enlargement of peritoneal lymph nodes. In other experiments, 0.1 ml packed metastatic tumour was transplanted into the subcutaneous site. The resultant tumours showed a similar growth pattern to s.c./s.c. transplants. Tumour extracts and the blood of tumour-bearing animals were screened for bacteriological contamination using standard microbiological techniques. In all cases these were found to be negative.

*Preparation of tumour cell suspensions*

Cell suspensions were prepared from 1° and 2° sites as follows. Primary tumour tissue was excised taking care to avoid any macroscopic contamination with host tissue. This material was roughly chopped, and any gross necrotic areas were separated off and discarded. Chopped pieces were washed twice with medium 199, twice with PBS, pH = 6.4, and 1–2 g were placed in 5 ml PBS, pH = 6.4, containing 0.2 mg ml$^{-1}$ collagenase (Sigma type II or Boehringer from *clos. histolyticium*). This mixture was stirred with a teflon coated metal bar for 15 min at 37°C in a glass universal on a magnetic stirring base (Gallenkamp) at setting 3. The cell suspension obtained from this treatment was discarded, 5 ml of fresh collagenase solution was added and the tumour pieces were stirred for a further 30 min under otherwise identical experimental conditions. These latter two steps were repeated and the cell suspensions from these two treatments were pooled. The method used to disaggregate metastatic (2°) tumour in the liver was slightly different to that used for the 1°. In this case, up to 3 g chopped tissue was treated with a single 15 ml aliquot of collagenase in PBS, pH = 7.1, for 45 min; otherwise the experimental conditions were identical to those used for the 1° tumours.

After disaggregation, undigested tissue was separated from single cells by filtration through Nylon Bolting Cloth (N6425N, Henry Simon Ltd., Stockport), and the single cells pelleted by sedimentation on a bench centrifuge. After three washes with 10 ml medium 199 + 2% new born calf serum, non-viable cells and erythrocytes were removed by layering 3 ml of the cell suspension ($3 \times 10^7$ cells) in medium 199 + 2% calf serum on 5 ml of a mixture (density = 1.08 g l$^{-1}$) containing 6.35% (w/v) Ficoll 400 (Pharmacia Fine Chemicals) and 9.97% (w/v) Hypaque (Winthrop Laboratories) in a universal and sedimenting at 1500 *g* for 15 min. After centrifugation, the viable cells were removed from the interface, diluted 1:4 with medium 199 + 2% calf serum, and washed three times with the same medium to remove the Ficoll/Hypaque. Prior to use, all cell suspensions were washed twice with medium 199.

A non-physiological pH was used in the disaggregation of 1° tumour tissue because it was previously found (Turner, 1979) that such conditions considerably increased the yield of single cells for this tumour line. In this current series of experiments, single cells were also prepared using collagenase at pH = 7.1; the results from these experiments indicated that the preparation of 1° cells at pH = 6.4 did not affect their growth characteristics *in vivo* or the surface properties that we investigated. Microscopic examination of purified 2° cell suspensions indicated that none of these were contaminated with hepatocytes. Although there must have been some host liver cells present initially, separate experiments on pieces of healthy liver showed that the particular disaggregation procedure employed disrupted most of the cells from this type of material, and any remaining cells were separated by the Ficoll/Hypaque treatment.

After purification, the final yields of 1° and 2° cells were, on average, $3.8 \times 10^7$ cells g$^{-1}$ tumour (s.d. $\pm 1.7 \times 10^7$ cells g$^{-1}$ tumour; 10 preparations) and $7.7 \times 10^7$ cells g$^{-1}$ tumour (s.d. $\pm 2.4 \times 10^7$ cells g$^{-1}$ tumour; 10 preparations) respectively. The initial yield of cells prior to purification was 2–4 times the final yield of cells. Primary cell preparations were 93.8% viable (s.d. ±1.8%; 10 preparations) as judged by Trypan blue exclusion and contained 8.8% (s.d. ±5.3%; 14 preparations) macrophages and 18.2% (s.d. ±6.5%; 14 preparations) polymorphonuclear leukocytes. Secondary cell preparations were 93.5% viable (s.d. ±2.2%; 10 preparations) as judged by Trypan blue exclusion and contained 1.7% (s.d. ±1.1%; 10 preparations) macrophages and 20.8% (s.d. ±6.1%; 10 preparations) polymorphonuclear leukocytes. Cell type analyses were determined by differential counts on smears stained with either Wrights or Giemsa/May Grunwald stains.

*Additional treatment of tumour cell suspensions*

In order to reduce the degree of macrophage contamination, some 1° cell suspensions (3 preparations) were further purified by allowing the cells to adhere to the surface of a plastic tissue culture flask. A suspension containing $2 \times 10^7$ cells in 15 ml RPMI 1640 (Flow Laboratories) + 10% (v/v) horse serum (Senior *et al.*, 1981) was placed in a 75 cm$^2$ plastic tissue culture flask (Nunc Gibco Europe Ltd.). The whole was equilibrated with an atmosphere of 5% $CO_2$ in air and incubated for 90 min at 37°C. The cells were transferred to another flask containing fresh medium, incubated for a further 30 min, and the latter two steps were repeated. Cells were washed twice with medium 199 prior to further experimentation. After this treatment, ~70% of the original cell number was recovered. Differential counts of Giemsa/May Grunwald stained smears indicated that the non-adherent cell preparations contained 20–25% of the number of macrophages originally present. The percentage of polymorphonuclear leukocytes present was not altered by this treatment.

In some experiments, 2° cells were suspended in a medium containing 90% (v/v) RPMI 1640 and 10% (v/v) calf serum. The whole was transferred to a 75 $cm^2$ plastic tissue culture flask, equilibrated under an atmosphere of 5% $CO_2$ in air, and incubated for 6 h at 37°C. After this treatment, the majority of cells were still in suspension, and therefore easily removed by centrifugation. This was followed by washing twice with medium 199.

The amount of neuraminidase-sensitive cell surface sialic acid was determined in 1° and 2° cell suspensions as follows. Preparations containing $4/5 \times 10^7$ cells in 1 ml PBS, pH = 7.1, were incubated with 0.2 units of neuraminidase (Type VIII, Sigma) for 1 h at 37°C with intermittent agitation to maintain the cells in suspension. Preliminary experiments indicated that this incubation time was optimum for the release of all the neuraminidase-sensitive sialic acid. After treatment, cells were removed by centrifugation, the neuraminidase was inactivated by standing at 60°C for 1 min and the supernatant was stored frozen until required for assay. A duplicate cell specimen was used to determine the protein content. The amount of sialic acid released was determined by the method described by Skoza & Mohos (1976).

*Control tissue and cell suspensions*

Specimens of liver were removed from healthy non-tumour bearing hamsters using normal surgical procedures. Tissue was chopped into small pieces and any gross blood contamination removed by extensive washing with PBS "A". Proteins were extracted immediately after washing.

The following control cell suspensions were prepared from healthy hamsters; macrophages, spleen cells, peripheral blood lymphocytes and polymorphonuclear leukocytes. Peritoneal macrophages (2 preparations) were washed out of the peritoneal cavity using two washes of 25 ml of medium 199 containing 50 units $ml^{-1}$ of heparin. This preparation was washed thrice with 10 ml medium 199 before use. Cells from 5 animals were pooled for each protein extraction, and the population was found to contain 72.3% macrophages by Giemsa/May-Grunwald staining.

Using two pairs of fine forcepts, cells were teased out of spleens (3 preparations) into 10 ml medium 199. Cell suspensions were filtered through nylon mesh and washed thrice with 10 ml medium 199. Any contaminating erythrocytes were removed using the Ficoll/Hypaque technique. Spleen cell preparations were found to contain 91.0% small lymphocytes as judged by the Giemsa/May-Grunwald technique. Peripheral blood lymphocytes were isolated as previously described (Freeman *et al.*, 1978).

Polymorphonuclear leukocytes (2 preparations) were induced by the i.p. injection of 40 mg oyster glycogen (BDH Chemicals) in 5 ml 0.9% (w/v) sodium chloride (Hirsch, 1956). After 3.5–4 h the induced cells were washed out of the peritoneal cavity using 25 ml medium 199. The suspension was washed thrice with medium 199 and further purified by subjecting $3 \times 10^7$ cells to centrifugation on a preformed gradient of Percoll ($1.02\,g\,l^{-1}$–$1.14\,g\,l^{-1}$; Pharmacia) for 20 min at 1500 *g*. The majority of the cells were present in a band at a mean density of $1.088\,g\,l^{-1}$. This latter density was higher than the value found for tumour polymorphs ($<1.08\,g\,l^{-1}$); the reason for this difference is being further investigated. The final cell preparation contained 89.9% polymorphs and 10.1% macrophages as judged by the Giemsa/May Grunwald technique. All non-tumour cell suspensions used were >90% viable as demonstrated by Trypan blue staining.

*Preparation of cell membranes*

Cell membranes were prepared from tumour tissue using a modified two-phase polymer (Polyethylene glycol 600 and Dextran T500) method. Membranes were prepared from tissue rather than isolated cells because preliminary studies had shown that very large numbers ($>2 \times 10^8$) of single cells were needed in order to obtain a satisfactory membrane yield. It was difficult, therefore, to obtain these numbers routinely without using tumours from different animals and processing pooled preparations. We did not want to use this approach because it would have made the data more difficult to interpret. Full details of the original method have been previously documented (Brunette & Till, 1971). Briefly, pieces of finely chopped tumour (2–3 g) were suspended for 5–10 min in 20 vol of ice-cold 10 mmol $l^{-1}$ Tris buffer, pH = 8.4, and homogenised using a glass Dounce homogeniser. This homogenate was subjected to a 30 sec spin at 1000 *g* and the subsequent supernatant was diluted with four volumes of PBS "A" and centrifuged at 12,000 *g* for 30 min. This crude membrane pellet was further purified by suspending in the two-phase polymer solutions, whereby the purified membrane appeared at the interface. All membrane preparations were washed thrice with PBS "A" before use.

Aliquots of membranes and cell homogenates were assayed for 5′ nucleotidase activity (Douglas *et al.*, 1972), acid phosphatase activity (Turner & Weiss, 1980) and DNA content (Munro & Fleck, 1966). The mean specific activity of 5′ nucleotidase from the membrane preparations was 3.5 $\mu$mol h$^{-1}$ mg$^{-1}$ protein, a value that was 5–6 times higher than the specific activity of this enzyme in the homogenate. The acid phosphatase and DNA content of the membrane preparations were <0.1% and 3–4% respectively of that measured in the homogenates. These values suggest that the lysosomal and nuclei contamination of our preparations were low. Furthermore, Brunette & Till (1971) reported that this method gave surface membranes with minimal contamination by smooth endoplasmic reticulum, mitochondria, and nuclei. We did not check for contamination by Golgi and it is possible that this type of contamination was present. The protein content of the membranes was, on average, 1.0 mg g$^{-1}$ tissue.

*Extraction of cell proteins*

Glycoproteins were extracted from pieces of viable tissue or single cell pellets or cell membrane preparations using a method similar to that described by Butters & Hughes (1974). Briefly, material to be extracted was suspended in approximately twice the volume of Tris/HCl, pH = 8.4 (10 mmol l$^{-1}$) buffer containing 0.5% (v/v) Triton X-100 and 1 mmol l$^{-1}$ phenyl methyl sulphonyl fluoride (PMSF). This mixture was left to stand at room temperature for 20 min. Unsolubilized material was removed by centrifugation at 600 *g* for 5 min, and the supernatant was stored at −20°C. The extract was further subjected to centrifugation (12,000 *g* for 4 min) prior to the estimation of the protein content of the extract by the Lowry method. According to Butters and Hughes, this extraction procedure removes the majority of glycoproteins associated with the cell membrane. Also, Bramwell and Harris (1978) reported that the glycoproteins extracted by SDS from a wide range of tumours were virtually identical to those extracted from corresponding isolated membranes.

*Electrophoresis*

The electrophoretic method and staining technique used were based on that described by Bramwell & Harris (1978). Prior to electrophoresis, cell extracts were diluted 1:2 vols. with Tris/HCl buffer, pH = 8.4 (10 mmol l$^{-1}$) containing 2% (w/v) sodium dodecyl sulphate (SDS), 10% glycerol, 0.1 mol l$^{-1}$ dithiothreitol and 0.001% (w/v) bromophenol blue. This mixture was immersed in a boiling water bath for 2 min, and left to cool at room temperature.

An aliquot of an extract (5–50 $\mu$l) containing 60 $\mu$g protein was separated by SDS gradient (7.5–20%) polyacrylamide gel electrophoresis in slabs. After electrophoresis, slab gels were stained with Coomassie Brilliant Blue R250 and destained in a solution of methanol/acetic acid/water. The following molecular weight markers were used to calibrate each electrophoretic separation: myosin, 200,000 daltons; phosphorylase B, 94,000 daltons (Sigma Chemical Co.); RNA polymerase subunits, 165,000, 155,000 and 39,000 daltons; bovine serum albumin, 68,000 daltons; and trypsin inhibitor (TI), 21,500 daltons (Boehringer).

*Preparation of labelled WGA*

Wheat germ agglutinin (WGA) was labelled with $^{125}$I using a modified procedure to that previously described (Bramwell & Harris, 1978). Briefly, 5 mg WGA (Calbiochem), 8 mg N-acetyl glucosamine and 2 $\mu$moles glucose were dissolved in 200 $\mu$l PBS "A" (Dulbecco's PBS-Ca and Mg free; Flow Labs.) contained in a 1.5 ml microfuge tube (720–690, Sarstedt). To this solution was added 50 $\mu$l lacto-peroxidase (5 mg ml$^{-1}$ in PBS "A"; L2005, Sigma), 20 $\mu$l glucose oxidase (1 mg ml$^{-1}$; Boehringer) and 1 mCi Na$^{125}$I (13–17 mCi $\mu$g$^{-1}$; Amersham). After mixing well, this mixture was left to stand for 30 min at room temperature with further mixing at 10 min intervals. The labelled lectin and free label were separated by passing the mixture down a small column containing 5 ml of packed Bio-Gel P6 (Biorad Labs.). The column was eluted using PBS "A" and the fractions that contained the labelled lectin were determined by the dropwise addition of saturated ammonium sulphate (~40% w/v). Over 90% of the original quantity of WGA was routinely recovered using this method for labelling the lectin. After preparation, the labelled lectin was dissolved in 100 ml 0.1 mmol l$^{-1}$ sodium phosphate buffer, pH = 6.8 (Gurr), containing 0.4 mmol l$^{-1}$ sodium chloride, 0.1% (w/v) sodium azide and 2.5 mg ml$^{-1}$ haemoglobin (Bovine Type II: Sigma) and stored at 4°C.

*Treatment of electrophoretic gels with labelled WGA*

Prior to treatment of slab gels, the fixed and stained gel was equilibrated in approximately 300 ml of the pH = 6.8 phosphate buffer containing 0.4 mmol l$^{-1}$ sodium chloride. This involved several changes over a period of 10 h with constant shaking. The equilibrated gel was incubated with the lectin solution at room temperature with constant shaking for 3–16 h, depending upon the specific radioactivity of the lectin. After treatment, unbound lectin was removed by washing for 2 days with approximately 10 changes of the phosphate buffer/sodium chloride solution. This was followed by a 30 min incubation in methanol/acetic acid/water (5:1:5). The gel was dried down by wrapping in dialysis membrane (Biorad) and leaving on a vacuum gel dryer overnight. Bound lectin was visualized by exposing the dried gel to no-screen X-ray film (Kodak, NS-2T) for 4–20 days. Autoradiographs were analysed using a scanning laser densitometer (LKB Instruments).

### Results

Figure 1 illustrates typical Coomassie Blue (CB) stained patterns and corresponding WGA autoradiographs after the separation of tumour extracts by electrophoresis in SDS gradient polyacrylamide gels. Tracks a, b, e and f are the CB patterns and tracks c, d, g and h are the same specimens respectively after the treatment of the gel with radiolabelled WGA and the measurement of the lectin binding by using autoradiography. It can be seen that the CB stained patterns for 1° and 2° preparations are very similar. Minor differences could sometimes be seen between 1° and 2° preparations, especially with tissue extracts, but visual examination and densitometric scans of the CB patterns from ten matched pairs of 1° and 2° cell preparations revealed no consistent differences. Any additional bands seen in tissue extracts probably arose from contamination with host material.

In contrast to the CB stained patterns, considerable differences were detected for the WGA binding patterns (see Figures 1 and 2). Four major bands were always detected on autoradiographs of 1° preparations; these are identified in Figure 2 by stars. It is clear that the expression of some of these bands is substantially reduced in extracts prepared

**Figure 1** Electrophoretic analyses of 1° and 2° tumour tissue (a–d) and tumour cell (e–h) extracts. Tracks a, b, e and f show patterns after Coomassie Blue staining and tracks c, d, g and h show the same specimens after the treatment of the gel with radioiodinated WGA and the detection of lectin binding using autoradiography. Mol. wt markers are indicated by arrows.

**Figure 2** Autoradiographs of WGA-binding to separated extracts from 5 matched-pairs of 1° and 2° cells. The position of the major bands are indicated by stars, and mol. wt markers by arrows.

from 2° tumour tissue or cells (Figure 1). Examination of extracts from 10 matched-pairs of 1° and 2° cells indicated that this reduction was a very consistent change and the data from 5 of these pairs are given in Figure 2 to illustrate this point. The approximate mol. wts of these major bands were estimated as 23,000, 84,000, 102,000 and 188,000 daltons.

Neuraminidase treatment of cells from the primary tumour released 0.72 $\mu$g sialic acid mg$^{-1}$ of protein (s.d. $\pm 0.17\,\mu g\,mg^{-1}$; 6 preparations), whereas similar treatment of cells from the metastatic tumour released 0.64 $\mu$g sialic acid mg$^{-1}$ protein(s.d. $\pm 0.15\,\mu g\,mg^{-1}$; 6 preparations). This difference was not significantly different by the Student's *t*-test ($P > 0.05$).

Figure 3 shows densitometric scans of autoradiographs. Results are given from three separate experiments in which extracts were prepared from 1° tissue, cells or membranes. The pattern for the glycoproteins isolated from membrane was very similar to that found for cells and tissue. One major difference was the absence of a band at 23,000 daltons in the membrane preparations. In addition, the binding of WGA to the separated membrane extracts was found to be 2–3 times greater than to material extracted from tissue or cells.

The WGA binding patterns of various control extracts are presented in Figure 4. Data are shown from two separate experiments. For the majority of specimens investigated, the patterns obtained were both different from, and weaker than, those observed for the 1° tumour cells. Data are not shown for liver tissue or peripheral blood lymphocytes because no significant WGA binding could be detected with extracts from liver, and the pattern for extracts from lymphocytes was very similar to that given for spleen cells. The binding to separated serum proteins, however, was strong and although one of these bands was in a similar position to a tumour band, the overall pattern for the serum proteins was very different. Figure 4 also illustrates the effect on the WGA pattern of removing adherent cells from 1° tumour cell preparations. Such treatment appeared to have no effect. Other control experiments were carried out in which 2° cells were incubated for up to 6 h at 37°C in growth medium. This type of treatment did not change the WGA binding pattern (data not shown).

Figure 5 demonstrates the effect on the WGA binding pattern of directly transplanting 1° tumour tissue into the liver, or transplanting 2° tumour tissue into the s.c. site. Also shown are the patterns for the original 1° and 2° tumour, and for the metastatic tumour resulting from the 2°/1° transplantation. These results clearly show that the detection of WGA bands is related to the site in which the tumour is growing, and not to the source of the tumour tissue.

**Figure 3** Densitometric tracings of autoradiographs of WGA binding to separated extracts from 1° tissue, cells and membranes. Data is presented from 3 experiments. Only 30$\mu$g protein were analysed from membrane extracts. Mol wt markers are indicated by arrows.

**Figure 4** Autoradiographs of the WGA-binding patterns obtained from the analysis of various non-cancerous specimens. Data is shown from two separate experiments; in each experiment the pattern of 1° tumour cells is given for comparison. Also shown (Tracks a and b) is the effect on the WGA-binding pattern of removing adherent cells from a 1° tumour cell preparation. In tracks d and e, the low mol. wt ends of the gel are missing; no bands were seen in these regions. Mol. wt markers are indicated by arrows.

**Figure 5** Autoradiographs of the WGA-binding patterns obtained from extracts of 1° and 2° tumour growing in different sites. (a) tumour growing in the liver after direct transplantation from the s.c. site. (b) tumour growing at s.c. site. (c) tumour growing in liver after metastasis from s.c. site. (d) tumour growing in s.c. site after transplantation of liver metastases. (e) liver metastases from (d). Mol. wt markers are indicated by arrows.

### Discussion

The current studies show that cells isolated from a local tumour express certain surface glycoproteins that are considerably reduced in cells isolated from the liver metastases. This was a consistent finding, although the extent of the reduction varied from animal to animal. Visual examination and densitometric scans of the Coomassie Blue stained patterns in conjunction with the WGA labelled patterns could not identify any major differences in protein composition between 1° and 2° preparations. This latter finding agrees with our previous investigations of surface proteins in this system using radioiodination (Guy *et al.*, 1979). This suggests that the cells in 2° tumours may not have lost a particular glycoprotein but that certain glycoproteins may have changed their degree of glycosylation.

It might be expected that any substantial changes in glycosylation of proteins would be reflected in changes in the electrophoretic mobility of the CB stained components. As such changes were not observed, it must be assumed that either the method we have used to analyse the protein composition is not sensitive enough to detect these changes or that the changes themselves are very small. Close inspection of the autoradiographs indicated that the WGA binding for some bands is very blurred and it is likely that these bands are composed of more than one component. Furthermore, the known microheterogeneity that can occur in the carbohydrate portion of glycoproteins could also contribute to this blurring. In order to further resolve the WGA bands, we have tried to apply smaller amounts of protein to the gel, but this approach has been unsuccessful.

In other studies, we have determined binding patterns using other lectins (Concanavalin A, RCA-60, Gorse and Peanut agglutinin). Patterns varied according to the lectin used; some being more complex than that obtained for WGA. From preliminary analyses, the only other lectin to give any consistent differences in 1° and 2° pattern is

Concanavalin A. The latter lectin appears to band strongly to many components in both 1° tumour and metastatic deposits, and where differences can be detected these seem to be minor in comparison with those observed for WGA. It seems that changes in WGA-binding glycoproteins involve a fairly distinct class of surface macromolecules. The results from these other studies will be the subject of a separate report.

WGA binds predominantly to N-acetylglucosamine residues of glycoproteins (Lis & Sharon, 1973), and we have shown that if an electrophoretically-separated 1° tumour extract is incubated with labelled WGA in the presence of 2% (w/v) N-acetylglucosamine, the degree of binding to the majority of the bands is substantially reduced (unpublished observations). On the other hand, WGA can also bind strongly to sialic acid (Bohvanadan & Katlic, 1979), a sugar that is present in large amounts in cell surface glycoproteins (Turner, 1982). Therefore, some of the observed differences between 1° and 2° may be due to reduced sialylation of surface glycoproteins on the 2° cells. If this is the case, then the content of sialic acid in certain surface glycoproteins must be more important than the total surface content, because we found that treatment of 1° and 2° cells with neuraminidase did not realise significantly different quantities of this sugar.

A number of other recent studies have detected differences in the surface properties of 1° and 2° cells. These include changes in antigenicity (Sugarbaker & Cohen, 1972; Fogel *et al.*, 1979; Schirrmacher *et al.*, 1982), cell agglutination (Price & Tarin, 1981), sensitivity to WGA cytotoxicity (Dennis *et al.*, 1981) and surface viscosity (Rivnay *et al.*, 1981).

It could be argued that the differences we observed between 1° and 2° tumours were due to differential contamination of the 1° tumour by host material. In an attempt to answer this question, we carried out analyses on a number of possible sources of cellular and tissue contamination but none of these gave WGA patterns that were similar to the pattern obtained for 1° tumour. Microscopic examination of stained cell preparations suggested that the major differences in cellular composition of 1° and 2° cell suspensions was the presence of more macrophages in the 1° preparations; although this contamination was small. We considerably reduced the level of macrophage contamination in 1° preparations by employing an adhesion technique. This type of treatment had little effect on the WGA pattern. On the other hand, Kaplan & Olstad (1981) have recently reported that macrophages treated with tumour ascitic fluid can express new glucosamine-containing glycoproteins. This latter observation suggests that some of our WGA binding could be due to macrophage contamination, but from our adhesion studies this would have to be a non-adherent population that was present in very low cell numbers. Additional studies, on isolated populations of tumour-activated macrophages, are currently in progress to try to resolve this problem.

Our results could also be explained by the presence of higher levels of hydrolytic enzymes in the 2° tumour preparations cleaving-off glycopeptides or carbohydrate groupings. At first sight, this might appear a feasible explanation; however, there are a number of observations that argue against it. First, during the procedure for preparing the extracts, an enzyme inhibitor, PMSF, was present. Second, incubation of purified 2° cells in medium plus calf serum at 37°C for 6 h did not result in the increased expression of the WGA bands. Previous radioiodination studies of the ML tumour demonstrated that under these conditions the cells synthesize surface macromolecules (Guy *et al.*, 1977). Third, the degrees of reduction in the expression of WGA bands were similar in extracts from corresponding 2° cells and tissue preparations. It might be expected that if enzymes were operating, this would not be true, as the tumour tissue was extracted immediately after excision whereas cells were not processed until two hours later. Fourth, if the activities of glycosidases were higher in metastatic cells or their extracts this would be reflected by reduced levels of the terminal sugars, fucose and sialic acid, in glycoproteins. This was not found to be the case. Measurements made on 1° and 2° cells indicated that they had similar amounts of neuraminidase-sensitive sialic acid. Also, the binding patterns of labelled Gorse lectin to separated extracts of 1° and 2° cells were virtually identical (unpublished observations). Gorse lectin has a high affinity for fucose residues.

Some previous studies with metastatic tumours have suggested that metastases arise from subpopulations of cells pre-existing in the local tumour; such cells having a special ability to carry out all the steps of the metastatic process (Post & Fidler, 1980). Therefore, the reduced WGA binding that we observed in liver metastases could reflect the emergence of a metastatic sub-population. In order to investigate this possibility, we injected metastatic tumour into the subcutaneous site. The tumour that arose expressed a WGA binding pattern that was indistinguishable from the original subcutaneous tumour pattern. Conversely, if subcutaneous tumour was injected directly into the liver, the WGA pattern was typical of normal 2° in the liver. These results do not provide evidence to support the cell selection hypothesis, but also they cannot exclude the possibility that selective processes are operating. The observed modulation of WGA binding when tumour is transplanted between different sites may reflect the emergence of different clonal subsets that have a high proliferative potential in a particular site.

Despite these difficulties in explaining our site-injection results in terms of a cellular mechanism, they do indicate that, in this tumour system, the site is very important in affecting surface properties. A similar conclusion has been reached by *in vivo* studies with other systems. Weiss & Harlos (1979) found that following direct injection of Walker ascites cells into other internal sites, the surface charge on the cells varied according to the site in which the tumour was growing. This change was only maintained if the tumour was passaged in this site. If the tumour was returned to the ascitic form, then the surface charge changed back to its original value. Rivnay *et al.* (1981) found that the plasma membrane microviscosity of Lewis Lung carcinoma cells was regulated by the site of tumour growth. Values were found to remain fairly constant as long as the tumour was maintained at the same site. When tumour cells were transferred to a new site, the cells acquired membrane viscosity typical of the new site.

The reason for cells from tumours having different surface properties when growing in different sites is unknown. As the site of tumour cell lodgement can affect local growth and spread (Keller, 1981; Schirrmacher *et al.*, 1982) and immunogenicity (Brooks *et al.*, 1981; Schirrmacher *et al.*, 1982), it might be speculated that there could be an association between these factors and the changes we have observed. Obviously, there are many other possible explanations and the clarification of this interesting phenomenon awaits further investigation.

We gratefully acknowledge the Department of Bacteriology, Royal Victoria Infirmary, Newcastle upon Tyne for carrying out the microbiological assays and the North of England Cancer Research Campaign for financial support.

**References**

BOHVANADAN, V.P. & KATLIC, A.W. (1979). The interactions of wheat germ agglutinin with sialoglycoproteins. The role of sialic acid. *J. Biol. Chem.*, **254**, 4000.

BRAMWELL, M.E. & HARRIS, H. (1978). An abnormal membrane glycoprotein associated with malignancy in a wide range of different tumours. *Proc. R. Soc. Lond. B.*, **201**, 87.

BROOKS, C.G., FLANNERY, G.R., WILLMOTT, N., AUSTIN, E.B., KENWRICK, S. & BALDWIN, R.W. (1981). Tumour cells in metastatic deposits with altered sensitivity to natural killer cells. *Int. J. Cancer*, **28**, 191.

BRUNETTE, D.M. & TILL, J.E. (1971). A rapid method for the isolation of L-cell surface membranes using an aqueous two-phase polymer system. *J. Membrane Biol.*, **5**, 215.

BUTTERS, T.D. & HUGHES, R.C. (1974). Solubilization and fractionation of glycoproteins and glycolipids of KB cell membranes. *Biochem. J.*, **140**, 469.

DENNIS, J., DONAGHUE, T., FLORIAN, M. & KERBEL, R.S. (1981). Apparent reversion of stable *in vitro* genetic markers detected in tumour cells from spontaneous metastases. *Nature*, **292**, 242.

DOUGLAS, A.P., KERLEY, R. & ISSELBACHER, K.J. (1972). Preparation and characterisation of the lateral and basal plasma membranes of the rat intestinal epithelial cell. *Biochem. J.*, **128**, 1329.

FOGEL, M., GORELIK, E., SEGAL, S. & FELDMAN, M. (1979). Differences in cell surface antigens of tumour metastases and those of the local tumour. *J. Natl Cancer Inst.*, **62**, 585.

FREEMAN, J.G., LATNER, A.L., SHENTON, B.K., TURNER, G.A. & VENABLES, C.W. (1978). TEEM test studies of effect of aprotinin on *in vitro* response of cancer patient lymphocytes to PPD. *Br. J. Cancer*, **38**, 636.

GUY, D., LATNER, A.L. & TURNER, G.A. (1977). Radioiodination studies of tumour cell surface proteins after different disaggregation procedures. *Br. J. Cancer*, **36**, 166.

GUY, D., LATNER, A.L. & TURNER, G.A. (1979). Surface protein distribution in cells isolated from solid tumours and their metastases. *Br. J. Cancer*, **40**, 634.

HIRSCH, J.G. (1956). Phagocytin: a bacterial substance from PMN's. *J. Exp. Med.*, **123**, 145.

KAPLAN, G. & OLSTAD, R. (1981). Heterogeneity in surface glycoproteins or mouse peritoneal macrophage populations. *Exp. Cell Res.*, **135**, 379.

KELLER, R. (1981). Induction of macroscopic metastases via surgery. The site of the primary tumour inoculation is critical. *Invn. Metast.*, **1**, 136.

LIS, H. & SHARON, N. (1973). The biochemistry of plant lectins (phytohemagglutinins). *Ann. Rev. Biochem.*, **42**, 541.

MUNRO, H.N. & FLECK, A. (1966). The determination of nucleic acids. *Meth. Biochem. Anal.*, **14**, 113.

POSTE, G. & FIDLER, I.J. (1980). The pathogenesis of cancer metastasis. *Nature*, **283**, 139.

PRICE, J.E. & TARIN, D. (1981). Lectin agglutinability of mammary tumours with differing metastatic colonisation potentials. *Differentiation*, **20**, 264.

RIVNAY, B., GORELIK, E., SEGAL, S. & SHINITZKY, M. (1981). Plasma membrane microviscosity of Lewis Lung carcinoma cells derived from local growth and pulmonary metastases. *Inv. Metast.*, **1**, 99.

ROOS, E. & DINGEMANS, K.P. (1979). Mechanisms of metastasis. *Biochim. Biophys. Acta.*, **560**, 135.

SCHIRRMACHER, V., FOGEL, M., RUSSMANN, E., BOSSLET, K., ALTEVOGT, P. & BECK, L. (1982). Antigenic variation in cancer metastasis: immune escape versus immune control. *Cancer Metast. Rev.*, **1**, 241.

SENIOR, R.M., CAMPBELL, E.J. & VILLINGER, B. (1981). Obtaining and culturing human and animal alveolar macrophages. In: *Methods for Studying Mononuclear Phagocytes*, (Eds. Adams *et al.*) New York: Academic Press. p. 69.

SKOZA, L. & MOHOS, S. (1976). Stable thiobarturic acid chromophore with dimethyl sulphoxide. Application to sialic acid assay in analytical de-o-acetylation. *Biochem. J.*, **159**, 457.

SUGARBAKER, E.V. & COHEN, A.M. (1972). Altered antigenicity in spontaneous pulmonary metastases from an antigenic murine sarcoma. *Surgery*, **72**, 155.

TARIN, D. (1982). Investigations of the mechanisms of metastatic spread of naturally occurring neoplasms. *Cancer Metast. Rev.*, **1**, 215.

TURNER, G.A. (1979). Increased release of tumour cells by collagenase at acid pH: A possible mechanism for metastasis. *Experientia*, **35**, 1657.

TURNER, G.A. (1982). Surface properties of the metastatic cell. *Inv. Metast.*, **2**, 197.

TURNER, G.A. & WEISS, L. (1980). Some effects of products from necrotic regions of tumours on the *in vitro* migration of cancer and peritoneal exudate cells. *Int. J. Cancer*, **26**, 247.

TURNER, G.A., GUY, D., LATNER, A.L. & SHERBET, G.V. (1980). Cell surface changes associated with the selection of spontaneous metastasis. In: *Metastasis: Clinical and Experimental Aspects*, (Eds. Hellmann *et al.*) The Hague: Nijhoff. p. 222.

WEISS, L. & HARLOS, J.P. (1979). Differences in the peripheries of Walker cancer cells growing in different sites in the rat. *Cancer Res.*, **39**, 2481.